# Congruent phylogenetic relationships of Melaphidina aphids (Aphididae: Eriosomatinae: Fordini) according to nuclear and mitochondrial DNA data with taxonomic implications on generic limits

**DOI:** 10.1371/journal.pone.0213181

**Published:** 2019-02-28

**Authors:** Zhumei Ren, Carol D. von Dohlen, A. J. Harris, Rebecca B. Dikow, Xu Su, Jun Wen

**Affiliations:** 1 School of Life Science, Shanxi University, Taiyuan, Shanxi, China; 2 Department of Biology, Utah State University, Logan, Utah, United States of America; 3 Department of Biology, Oberlin College and Conservatory, Oberlin, Ohio; 4 Data Science Lab, Office of the Chief Information Officer, Smithsonian Institution, Washington, DC, United States of America; 5 Key Laboratory of Medicinal Animal and Plant Resources of the Qinghai-Tibetan Plateau in Qinghai Province, School of Life Science, Qinghai Normal University, Xining, Qinghai, China; 6 Department of Botany MRC-166, National Museum of Natural History, Smithsonian Institution, Washington, DC, United States of America; Sichuan University, CHINA

## Abstract

Melaphidina aphids (*Rhus*-gall aphids; Eriosomatinae: Fordini) comprise five genera from eastern Asia and one monotypic genus from eastern North America. Melaphidina are unique in feeding on plant species of *Rhus* subgenus *Rhus* (Anacardiaceae), on which they form galls during the summer. The phylogenetic relationships among some species of Melaphidina aphids remain controversial. In this study, we sought to resolve the backbone phylogeny of Melaphidina aphids by sampling 15 accessions representing all six genera, all species, and all subspecies except *Meitanaphis microgallis* using 20 gene regions: five nuclear genes as well as 13 protein-coding genes and two rRNA genes of the mitochondrial genome. Phylogenetic analyses included Bayesian and maximum likelihood methods. Independent analyses of nuclear and mitochondrial genes returned congruent topologies, and analyses of all gene regions combined showed well-supported relationships among Melaphidina species. In particular, these were: (1) *Nurudea* (excluding *N*. *ibofushi*) is sister to a clade composed of the five remaining genera; (2) the monotypic North American genus *Melaphis* is sister to a clade comprising the four remaining genera; and (3) (*Schlechtendalia* + *N*. *ibofushi*) is sister to the clade (*Floraphis* (*Meitanaphis* + *Kaburagia*). Our results support the transfer of *Meitanaphis flavogallis* to *Kaburagia* as an additional subspecies or species, and the recognition of *Floraphis* as a distinct genus. This study provides important molecular resources for subsequent evolutionary studies using more nuclear genes on the Melaphidina aphids and their close allies.

## Introduction

*Rhus*-gall aphids (Melaphidina) feed on the developing shoots or leaves of sumac, namely, species of *Rhus* subgenus *Rhus* (Anacardiaceae), on which they induce galls. The galls are called *wu-bei-zi* in China and are important in traditional medicine, rubber production, and improving leather quality because of their rich tannin contents [1–3]. *Rhus*-gall aphids were formerly placed in the subtribe Melaphidina within the tribe Fordini by Heie [4], Blackman & Eastop [5], and Remaudière & Remaudière [6], but they were later raised to tribe Melaphidini by Zhang et al. [1] and Heie & Wegierek [7]. The most current classification synonymizes Melaphidini with Fordini, without additional subtribal recognition [8]. Nevertheless, the Melaphidina aphids form a well-supported clade within Fordini according to recent molecular phylogenetic analyses [9–10]. Melaphidina aphids traditionally comprised six genera: *Floraphis*, *Kaburagia*, *Melaphis*, *Meitanaphis*, *Nurudea* and *Schlechtendalia* [1,11], although some disagreements have arisen over whether *Floraphis* and *Meitanaphis* comprise genera distinct from *Nurudea* and *Schlechtendalia*, respectively [10,12] or not [8]. In this study, we follow the generic delimitation of Eastop & Hille Ris Lambers [11] and Zhang et al. [1] and refer to six genera of Melaphidina aphids for the convenience of discussion.

Melaphidina aphids exhibit a classical eastern Asian—eastern North American biogeographic disjunction, which is common among many groups of plants and animals [13–21]. Melaphidina has the greatest diversity in eastern Asia, while only one monotypic genus (*Melaphis*) is native to North America [1,11,22–23]. Melaphidina also have complex life cycles with cyclical parthenogenesis over multiple generations, which sequentially feed on sumac plants as primary hosts in summer and mosses as secondary hosts in winter [1–2,24]. The aphid-sumac-moss association is unique and represents a potential model for studying species interactions across kingdoms within the context of the biogeographic disjunction, which occurs similarly in both Melaphidina and their sumac hosts [9,12,25]. However, the utility of this system for biogeographic and co-evolutionary research has been incompletely realized due to limited by poorly resolved phylogenetic relationships within Melaphidina.

Prior studies on the phylogeny of Melaphidina have been largely constrained by limited sampling or have failed to find high support for relationships among the genera and species. Two prior studies focused on resolving phylogenetic relationships within the family Aphididae and the tribe Fordini, respectively, from the nuclear *EF-1*α gene [26] and *EF-1*α and mitochondrial *COI* gene [27]. These studies sampled only three of six genera of Melaphidina. Two additional studies focused on Melaphidina specifically, but showed low support for relationships among the genera [9,12]. Li et al. [10] investigated the monophyly of the subfamily Eriosomatinae, including nine Melaphidina species, and Bayesian inference supported Melaphidina monophyly and generic relationships, but MP and ML analyses were less supportive. Recently, Ren et al. [28] investigated the evolutionary relationships within Melaphidina using sequences of the complete mitochondrial genome, which revealed relatively strong support at many internal nodes but could not resolve the position of the North American *Melaphis* with high support across all analyses. Therefore, additional work on the phylogeny of Melaphidina using nuclear data is needed.

The primary objectives of this study were to (1) further test the phylogenetic relationships of all six genera of Melaphidina aphids using five nuclear genes combined with the 13 protein-coding genes and two rRNA genes of mitochondrial genomes; and (2) explore the taxonomic implications for Melaphidina aphids in light of the phylogenetic framework, especially concerning the generic limits. Our sampling of nuclear genes comprised *Elongation factor 1 alpha* (EF-1α), *Histone H3* (H3), *Wingless* (WG), 18S ribosomal RNA (18S rRNA) and *long—wavelength rhodopsin* (LWO), which we obtained by genome skimming via low- to high-density shotgun sequencing of total genomic DNA [29].

## Results

Altogether, the five nuclear genes and 15 mitochondrial genes represented 19,371 characters, of which 5,614 (29.0%) were polymorphic, and 3,709 (19.1%) were parsimony-informative (Table 1). Independently, the protein-coding genes of the mitochondrial genome had a concatenated length of 10,988 bp in length and the two ribosomal RNA genes comprised 2,081bp. The five nuclear genes had more variable sites than the mitochondrial genes, mainly distributed in the intron regions.

**Table 1:** Collection information for the Melaphidina aphid samples and outgroups used in this study. All the aphid specimens were alate viviparous females and we identified them according to the taxonomy of Zhang et al. [1]. All the samples were collected from China except for *Melaphis rhois* from United States of America and deposited at the School of Life Science, Shanxi University, China.

Species or subspecies	Voucher	Location	GenBank accession					
			Mitochondrion	EF-1α	WG	H3	LWO	18S
*Floraphis meitanensis*	*Ren A118*	Sangzhi, Hunan	MF043990	MF152698	MF159567	MF152704	MF179854	MF152689
*Floraphis choui*	*Ren A403*	Hanzhong, Shaanxi	MF043980	MF152697	MF159566	MF152703	MF179853	MF152688
*Kaburagia rhusicola ensigallis*	*Ren A1126*	Zhushan, Hubei	MF043984	MF152699	MF159568	MF152705	MF179859	MF152690
*Kaburagia rhusicola ovogallis*	*Ren A174*	Yuncheng, Shanxi	MF043986	MF159561	MF159569	MF159564	MF179860	MF152691
*Kaburagia rhusicola ovatirhusicola*	*Ren A1513*	Huozhou, Shanxi	MF043985	MK424019	MK412328	MK412079	MK412094	MF280268
*Kaburagia rhusicola rhusicola*	*Ren A1539*	Huozhou, Shanxi	MF043987	MK424021	MK412329	MK412080	MK412095	MF280269
*Meitanaphis elongallis*	*Ren A250*	Chenggu, Shaanxi	MF043989	MF152700	MF159570	MF152706	MF179855	MF152692
*Meitanaphis flavogallis*	*Ren A2012*	Emei, Sichuan	MF043982	MK424022	MK412327	MK412081	MK412096	MF280270
*Melaphis rhois*	*Ren A3037*	Ohio, Columbus	KY624581	MF159562	MF159571	MF152707	-	MF152693
*Nurudea shiraii*	*Ren A184*	Malipo, Yunnan	MF043978	MF152701	MF159572	MF152708	MF179856	MF152694
*Nurudea ibofushi*	*Ren A1796*	Wufeng, Hubei	MF043981	MK424020	MK412332	MK412082	MK412097	MF280271
*Nurudea yanoniella*	*Ren A267*	Chenggu, Shaanxi	MF043983	MK424024	MK412331	MK412083	MK412098	MF280273
*Nurudea yanoniella*	*Ren A1677*	Yangxian, Shaanxi	MK435595	MK424023	MK412330	MK412084	MK412099	MK424018
*Schlechtendalia chinensis*	*Ren A1798*	Wufeng, Hubei	KX852297	KF601635	MK412326	-	MF179857	-
*Schlechtendalia peitan*	*Ren A242*	Wufeng, Hubei	MF043979	MF159563	MF159573	MF152709	MF179858	MF152695
*Baizongia pistaciae*	*Ren A313*	Wufeng, Hubei	MF043988	MF152696	MF159565	MF152702	-	MF152687

Independent analyses of each of the five nuclear genes yielded topologies with high support for clades, especially comprising species within the same genus, but the relationships among deep nodes had low support (e.g., ML bootstrap < 60%). Nevertheless, the analyses of five concatenated nuclear genes with indels coded as new characters resulted in the highly supported topologies that were the same as the mitochondrial BI and ML trees.

**Fig 1:**
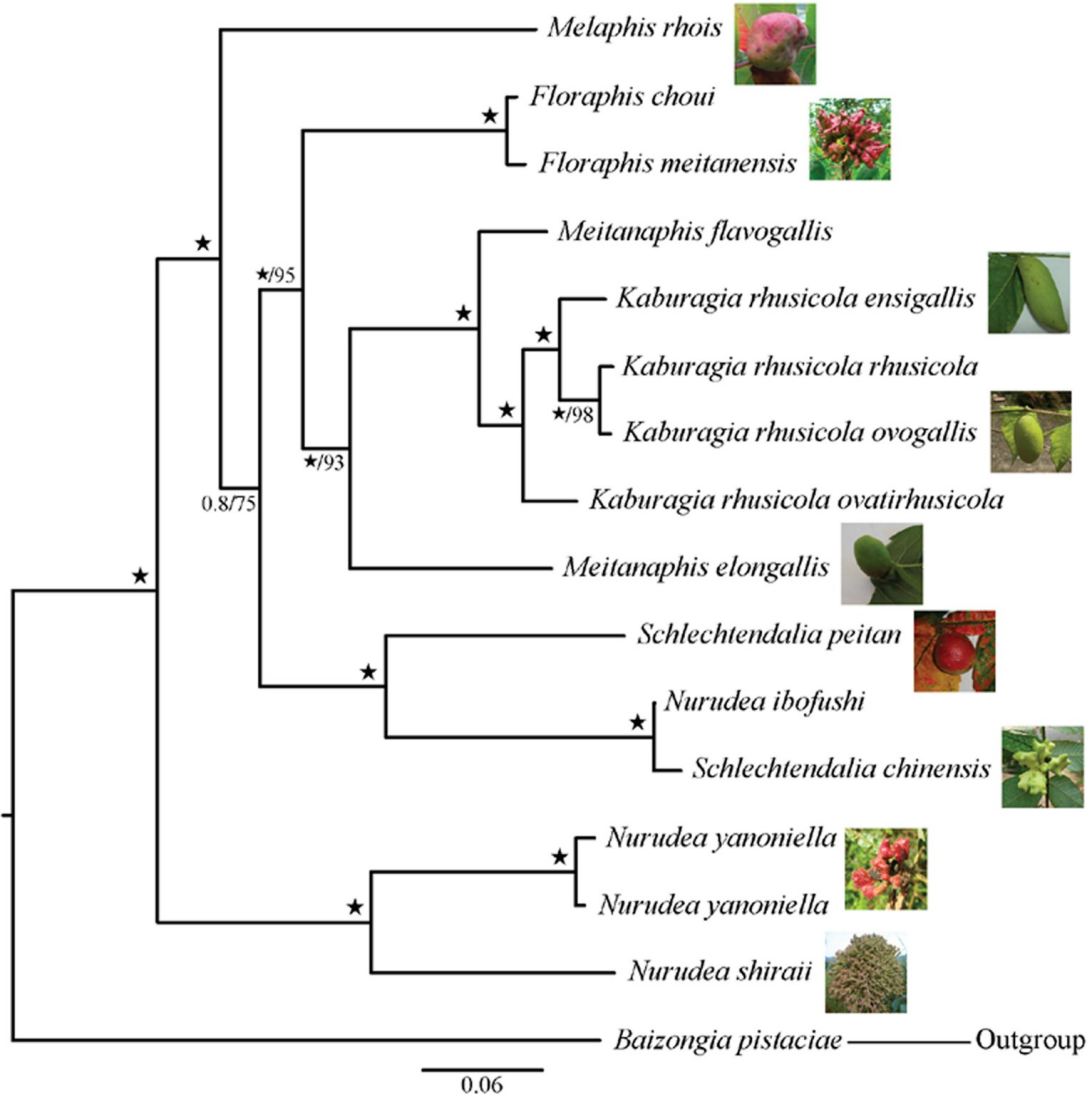
Bayesian 50% majority-rule consensus tree of the Melaphidina aphids based on the combined dataset of 15 mitochondrial and five nuclear gene sequences. Numbers on the branches show the Bayesian posterior probabilities (PP, left) and bootstrap values from maximum likelihood (BS, right) analyses. Stars represent nodes with 1.00 PP and 100% BS.

We concatenated the 15 mtDNA genes with the five nuclear datasets based on the results of an ILD test (P = 0.38 > 0.01). The concatenated dataset yielded ML and BI topologies that were congruent with topologies obtained from analyses of the 15 mtDNA genes and, independently, the concatenation of the five nuclear genes (Fig 1).

The concatenated nuclear and mitochondrial dataset showed that the six genera of Melaphidina aphids composed five generally well-supported clades: *Nurudea*, *Melaphis*, *Schlechtendalia*, *Floraphis*, and *Meitanaphis* + *Kaburagia*. The topology supported *Nurudea* except *N*. *ibofushi* as sister to a clade of the remaining species with high support (ML-BS = 100%, BI-PP = 1.00), and the North American *Melaphis* was sister to a clade of *Schlechtendalia* + *N*. *ibofushi*, *Floraphis*, and *Meitanaphis* + *Kaburagia* (ML-BS = 99%, BI-PP = 1.00). *Schlechtendalia* + *N*. *ibofushi* was sister to the clade (*Floraphis* (*Meitanaphis* + *Kaburagia*)) with low support (BI-PP = 0.8 < 0.90, ML-BS = 75%). *Kaburagia* + *Meitanaphis* formed a clade with ML-BS = 93% and PP = 1.00 that was sister to *Floraphis* (BS = 95% for RAxML analysis, BI-PP = 1.00).

## Discussion

The phylogenetic analyses of concatenated nuclear and mitochondrial data show well-resolved relationships among all species across the six Melaphidina genera: (1) *Nurudea* (excluding *N*. *ibofushi*) is sister to a clade composed of all other genera; (2) the monotypic North American genus *Melaphis* is sister to the clade comprising the other four genera; (3) the *Schlechtendalia* + *N*. *ibofushi* clade is sister to the clade of *Floraphis* (*Meitanaphis* + *Kaburagia*); (4) *Kaburagia* and *Meitanaphis* from eastern Asia comprise a clade that is sister to *Floraphis*; and (5) *Meitanaphis* is paraphyletic, with *M*. *flavogallis* sister to the clade of four subspecies of *Kaburagia*, and its type species, *M*. *elongallis*, is sister to the *Meitanaphis flavogallis + Kaburagia* clade.

The well-resolved phylogeny provides important insights into the generic limits of Melaphidina aphids. Our results strongly support recognizing *Floraphis* Tsai & Tang as a distinct genus [30]. *Floraphis* was synonymized with *Nurudea* Matsumura by Eastop & Hille Ris Lambers [11], and this treatment was accepted by Blackman & Eastop [22], Remaudière & Remaudière [6] and Favret [8]. However, Zhang et al. [1] maintained *Floraphis* as a separate genus on the basis of the numbers of antennal segments, presence or absence of stigma on the forewing, host plant preferences, and gall shapes. Previous molecular studies have also supported the recognition of *Floraphis* [9,12]. However, the phylogenetic position of *Floraphis* has not been well resolved. It was placed as sister to *Melaphis* with low support values (PP = 0.77, BS < 50% [12]; PP = 0.83 [9]). A recent analysis using 15 mitochondrial genes supported *Floraphis* as sister to the *Kaburagia* + *Meitanaphis* [28], as does our current phylogeny using five nuclear genes in addition to the mitochondrial data. Thus, the accumulating evidence is converging on a consensus of *Floraphis* as distinct from *Nurudea* and as sister to the *Meitanaphis* + *Kaburagia* clade.

*Meitanaphis* was erected by Tsai & Tang [30] with *M*. *elongallis* as type species. Tang [31] and Xiang [32] described two new species, *M*. *flavogallis* and *M*. *microgallis*, on *Rhus punjabensis* and *R*. *potaninii*, respectively. Past studies [12,28], as well as our current study, show that *Meitanaphis* is paraphyletic with its type species *M*. *elongallis* [30] as sister to a clade consisting of *M*. *flavogallis* and *Kaburagia*, and *M*. *flavogallis* sister to the clade of four *Kaburagia* subspecies. Yang et al. [33] noted that the antennal characters of *M*. *elongallis* are very distinct from other Melaphidina genera, whereas those of *M*. *flavogallis* are very similar to *Kaburagia* species. These authors suggested that *Meitanaphis* be revised and recommended that *M*. *flavogallis* be transferred to *Kaburagia*, thus rendering both *Kaburagia* and *Meitanaphis* monophyletic [33]. Our results also show *Meitanaphis* to be paraphyletic, with type species *M*. *elongallis* [30] sister to a clade consisting of *M*. *flavogallis* and *Kaburagia*, and with *M*. *flavogallis* sister to the clade of the four *Kaburagia* subspecies. *Meitanaphis flavogallis* could be transferred to *Kaburagia* as a new subspecies or as a species sister to the four *Kaburagia* subspecies. A comprehensive analysis of all diagnostic morphological characters, as well as sequence data, from wider population sampling of all *Kaburagia* and *Meitanaphis* subspecies/species is called for to resolve the taxonomy of *Meitanaphis*, as well as *Kaburagia*. Additionally, *Meitanaphis* has previously been considered as a synonym of *Schlechtendalia* [8,11]. However, this is clearly rejected by our results and prior studies, which show generic-level distances between *Meitanaphis* and *Schlechtendalia* and support them as distinct genera [10,12,28].

The monophyly of *Kaburagia* Takagi has been challenged recently by molecular data [12]. *Kaburagia* was erected based on commercial galls exported from China [34] and currently contains four subspecies *sensu* Zhang et al. [1]. The four subspecies are distinguished on the basis of minor differences in the number of wax glands on the dorsum, tarsal I chaetotaxy, number of setae on the cauda, host plants, and gall shapes. Yang et al. [12] sampled the four subspecies of *Kaburagia* and three species of *Meitanaphis* and found *Kaburagia* to be paraphyletic with respect to *M*. *flavogallis* and *M*. *microgallis*. In contrast, Zhang & Qiao [26] and Ren et al. [9] found that the four subspecies of *Kaburagia* were monophyletic, but both of these studies included only the type species, *M*. *elongallis*. Our present study grouped the four *Kaburagia* subspecies with high support as a clade, to which *M*. *flavogallis* formed a sister relationship. The status of *M*. *microgallis* needs to be tested with additional samples and data.

Prior molecular studies have also uncovered taxonomic problems in *Nurudea* Matsumura. Zhang et al. [1] proposed a taxonomic treatment of *Nurudea* comprising three species: *N*. *ibofushi*, *N*. *shiraii* and *N*. *yanoniella*. Two species, *N*. *shiraii* and *N*. *yanoniella*, were included in Yang et al. [12], who found that the species formed a monophyletic group that was sister to the remaining Melaphidina. Ren et al. [9] included all three species and found that *Nurudea* was paraphyletic: *N*. *shiraii* and *N*. *yanoniella* formed a clade, but *N*. *ibofushi* was sister to *Schlechtendalia chinensis* and genetically very similar to that species. However, Ren et al. [9] utilized only three mitochondrial genes (*COI*, *COII* and *Cytb*) and a single nuclear gene (*EF-1α*). Our current analyses, which add evidence from more genes, replicate the findings of previous studies showing *Nurudea* to be paraphyletic. *N*. *ibofushi* may be best transferred to *Schlechtendalia*, where it could be classified as a subspecies of *S*. *chinensis* or a species very closely related to it [9,28]. However, further studies are needed to test the monophyly of *Nurudea* with broad sampling in both China and Japan before any formal taxonomic revision is made for the genus.

As currently classified, the monophyly of *Schlechtendalia* Lichtenstein is confirmed by molecular evidence. Bell [35] was first to describe aphid species forming galls on sumac leaves in China as *Aphis chinensis*. Lichtenstein [36] established the genus *Schlechtendalia* and transferred *Aphis chinensis* to it. The North American species, *Melaphis rhois*, was originally considered to represent a western population of *S*. *chinensis*. Subsequently, *M*. *rhois* experienced complicated nomenclatural turnover and was given names including *Pemphigus sinensis* Walker, *Byrsocrypta rhois* Fitch, and *Melaphis chinensis* Baker. Eastop & Hille Ris Lambers [11] treated *Melaphis* and *Schlechtendalia* as different genera, with species from China and North America assigned to *S*. *chinensis* and *M*. *rhois*, respectively. More recently, the circumscription of *Melaphis* and *Schlechtendalia* as independent genera has been supported by morphological and molecular evidence [1,9,12,28]. Our present study strongly supports recognition of *Melaphis rhois* and *Schlechtendalia* as distinct genera that are distantly related. The North American *Melaphis rhois* is sister to a clade comprising *Schlechtendalia*, *Floraphis*, and *Meitanaphis* + *Kaburagia*.

Here, we present a well-resolved phylogeny showing relationships among the genera of Melaphidina aphids. However, more extensive taxon sampling at the population, subspecies, and species levels, and incorporation of both morphological and ecological characters as well as additional molecular data are necessary to construct a thorough taxonomic revision of the group. With additional new data, we should be able to test among alternative hypotheses of relationships and revise the taxonomy of Melaphidina with greater certainty.

## Materials and methods

### Ethics statement

All the samples of Melaphidina aphids employed in this study were collected from the sumac galls that are not endangered, and these trees grow in public field where no permission for collection of leaves is needed.

### Taxon sampling and DNA sequences

We collected live samples of species of Melaphidina aphids from mature, fresh sumac galls in the field (Table 1). From the galls, we extracted multiple individual aphids, which were genetically identical due to parthenogenetic development. We stored some of the collected individuals in 75% ethanol for taxonomic identification using microscopy and others in 100% ethanol for DNA extraction. We sampled from 15 accessions, which represented eleven species, including four subspecies, and all six genera of the Melaphidina aphids. We also sampled the closely related aphids, *Baizongia pistaciae*, from the tribe Fordini as an outgroup [26–27,37–39]. We deposited the voucher specimens at the School of Life Science of Shanxi University in China.

We extracted genomic DNAs using five individuals from the same gall with the DNeasy extraction kits (QIAGEN, Valencia, CA). We sent the DNAs to the Genomic Sequencing and Analysis Facility (GSAF), University of Texas, Austin for library construction and sequencing. Paired-end reads of 2x150 bp were generated on an Illumina NextSeq 500 platform with an insert size of 400 bp. We utilized the shotgun reads from genome skimming to obtain the five nuclear markers, 18S, *EF-1α*, H3, WG, and LWO by first mapping the reads to alignments of available sequences on GenBank and then performing *de novo* assemblies of reads in SPAdes [40]. We submitted the newly generated sequences of the nuclear markers to GenBank (Table 1). The accession numbers of the complete mitochondrial genome, from which the sequences of the 13 mitochondrial protein-coding genes and two rRNA genes were available, are also shown in Table 1.

### Phylogenetic analysis

We performed phylogenetic analyses using five nuclear genes and 15 mitochondrial genes with separate and concatenated sequences. We aligned sequences using MAFFT v7.017 [41–42] implemented in Geneious 10 with default settings (http://www.geneious.com) [43], which allow for auto-selection among MAFFT algorithms for alignment based on data size. After alignment, we omitted highly variable regions within introns of nuclear genes before further analyses.

The protein-coding genes of the mitochondrial genome in species of Melaphidina are conserved in length with only a few gaps. Therefore, we coded indels only for the two mitochondrial rRNA genes and nuclear genes. We coded the indels as binary characters using the simple coding method of Simmons and Ochoterena [44] in SeqState [45]. We employed Sequence Matrix v1.8 [46] to combine the DNA data and binary characters.

For model-based analyses (see below), we set models of evolution according to results from jModelTest v.2.1.7 [47–48]. JModelTest resolved the best-fit models under the corrected Akaike Information Criterion (AICc), and we selected the highest-scoring models implemented in MrBayes: GTR + I + G model for *COI*, *COII*, *COIII*, *ATP6*, *ND1*, *ND2*, *ND4*, *ND5*, *Cytb*, *WG*, *LWO* and *16S rRNA* genes; GTR + G model for *ATP8*, *12S rRNA*, 18S, H3 and *EF-1α*; GTR + I model for *ND3*, *ND4L* and *ND6* genes. We performed Bayesian phylogenetic analyses (BI) in MrBayes v.3.2.5 [49–50] for all individual genes, the combined nuclear genes, and the combined mitochondrial genes. We concatenated the mitochondrial genes because they are maternally inherited and represent a non-recombining locus. We also concatenated all the mitochondrial and nuclear genes based on the outcome of an incongruence length difference (ILD) test [51] in PAUP* [52]. For Bayesian analyses of individual genes, we treated binary indel codes as a separate partition, and performed two independent, simultaneous runs of the Markov Chain Monte Carlo (MCMC) for 10,000,000 generations starting from different random trees. We applied three hot and one cold chains for each run and sampled the cold chain every 1000 generations. We removed a burn-in of 2,500 trees, or 25%, and used the remaining trees to construct 50% majority-rule consensus trees to show posterior probabilities (PP) of clades; 50% majority-rules trees were visualized in FigTree v1.4.2 (http://tree.bio.ed.ac.uk/software/figtree/). We analyzed the concatenated dataset in MrBayes with partitions for each gene and for the binary indel codes. We applied unlinked model parameters to each partition. The model for the coded binary partitions was a default Standard Discrete Model in MrBayes [50].

We also conducted the maximum likelihood (ML) analyses using RAxML v.8.2 [53] with the same data partitioning as in the BI analysis. We selected the GTR continuous gamma model and performed bootstrapping with a random-number seed and 1000 replicates [54–55].
